# Heating Homes with Servers: Workload Scheduling for Heat Reuse in Distributed Data Centers

**DOI:** 10.3390/s21082879

**Published:** 2021-04-20

**Authors:** Marcel Antal, Andrei-Alexandru Cristea, Victor-Alexandru Pădurean, Tudor Cioara, Ionut Anghel, Claudia Antal (Pop), Ioan Salomie, Nicolas Saintherant

**Affiliations:** 1 Computer Science Department, Technical University of Cluj-Napoca, Memorandumului 28, 400114 Cluj-Napoca, Romania; marcel.antal@cs.utcluj.ro (M.A.); victor.padurean@student.utcluj.ro (V.-A.P.); ionut.anghel@cs.utcluj.ro (I.A.); claudia.pop@cs.utcluj.ro (C.A.); ioan.salomie@cs.utcluj.ro (I.S.); 2 Physics Department, Merton College, Merton St, Oxford OX1 4JD, UK; andrei-alexandru.cristea@merton.ox.ac.uk; 3 Qarnot Computing, 40–42 Rue Barbès, 92120 Montrouge, France; nicolas.saintherant@qarnot-computing.com

**Keywords:** heat reuse, distributed data centers, workload scheduling, machine learning, mathematical modeling

## Abstract

Data centers consume lots of energy to execute their computational workload and generate heat that is mostly wasted. In this paper, we address this problem by considering heat reuse in the case of a distributed data center that features IT equipment (i.e., servers) installed in residential homes to be used as a primary source of heat. We propose a workload scheduling solution for distributed data centers based on a constraint satisfaction model to optimally allocate workload on servers to reach and maintain the desired home temperature setpoint by reusing residual heat. We have defined two models to correlate the heat demand with the amount of workload to be executed by the servers: a mathematical model derived from thermodynamic laws calibrated with monitored data and a machine learning model able to predict the amount of workload to be executed by a server to reach a desired ambient temperature setpoint. The proposed solution was validated using the monitored data of an operational distributed data center. The server heat and power demand mathematical model achieve a correlation accuracy of 11.98% while in the case of machine learning models, the best correlation accuracy of 4.74% is obtained for a Gradient Boosting Regressor algorithm. Also, our solution manages to distribute the workload so that the temperature setpoint is met in a reasonable time, while the server power demand is accurately following the heat demand.

## 1. Introduction

Data centers (DCs) are consuming about 2–3% of the total electrical energy generated worldwide, thus, they are becoming a global problem. The high energy demand is used not only for the DCs’ primary business objective, which is to execute their client’s workload, but also to maintain temperature conditions for the safe operation of IT equipment. As result, dealing with excess heat has become an expensive process that is negatively affecting the DCs’ profit and sustainability [1]. The continuous hardware upgrades of computing resources that are increasing the power density of the processor make the cooling processes even more complex. This generated even higher energy demands for cooling systems in an effort to remove the heat produced by the computing resources. Studies show that DCs in 2019 consumed over 1900 MW of energy, while the associated heat generated is sufficient to heat around 2 million households need 20 GJ of heating on average [2]. 

In current DCs, the cooling processes are executed continuously to remove the heat generated by the computing resources and transfer it to a heat exchanger that uses air or liquid coolant [3]. This is rather inefficient, as the energy is consumed twice: first by the computing resources to execute the clients’ workload and second by the cooling system to dissipate the accumulated heat. To address this issue, new research directions have emerged lately aiming to reuse the otherwise wasted heat of the DCs in nearby district heat grids [4,5,6]. 

Despite these recent efforts, only a few DCs are effectively reusing the generated heat and only a fraction of the excess heat is being recovered [7,8]. There are several reasons making heat reuse difficult. First, the DCs should be in an urban agglomeration benefiting from policies, operations, and infrastructure that enable the smart distribution of thermal energy. Few cities are offering the needed conditions for DCs to re-use their heat. A positive example is Stockholm where almost 10 percent of the city’s heating needs are assured by using heat recovery [9,10]. Second is the relatively low quality of the recovered heat and the losses that occur when it is transported over long distances. To cope with this problem, systems were designed to transfer the absorbed heat at a higher temperature, making it suitable for long-distance transportation. Heat pumps are used to increase the temperature of the recovered heat to make it more marketable. With the help of heat pumps, the heat generated by servers at around 40 degrees Celsius can be transferred to heat water to around 80 degrees Celsius, suited for long-distance transportation in the nearby residences. At the same time, studies have shown that the coolant flow rate and server room outlet temperatures are important factors for the system’s overall efficiency. The third is the concern regarding the exploitation of DCs’ thermal flexibility of the server rooms in safe conditions for the IT equipment’s operation [11]. The equipment can overheat leading to malfunctions if the temperature within the server room increases to generate more heat. The formation of hot spots needs to be prevented using complex management strategies and accurate simulations of the thermodynamic processes within the DC are used to avoid dangerous hot spots [12,13]. 

**Figure 1:**
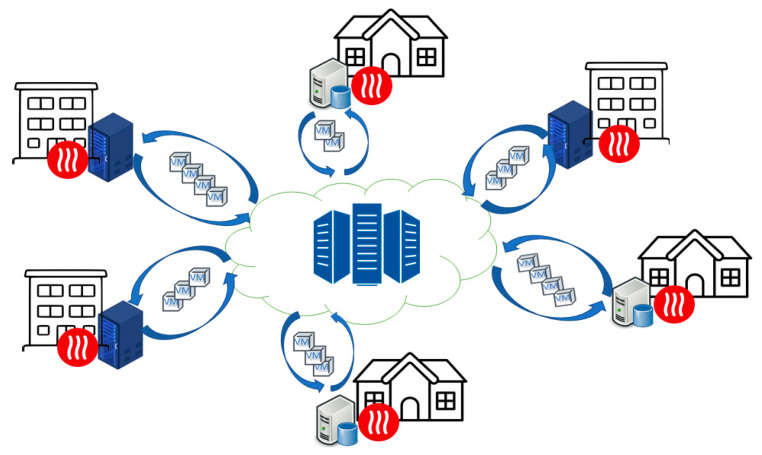
Distributed DC and computing power-based heating.

In this paper, we address the problem of DCs heat reuse from a novel perspective while considering the case of distributed DCs. In such a design, the IT equipment is not deployed in a server room but is distributed and deployed into buildings and used to provide heat for the tenants while executing the workload (see Figure 1). 

The aforementioned design can be more energy efficient because it will also reuse the electricity that is normally used for space heating for executing the workload. The IT equipment is used as a primary source of heat being deployed in the building’s rooms, which eliminates the costs associated with the cooling processes. The building network connection is used to get the delay-tolerant workload to be executed.

In summary, the paper provides the following contributions:

- Definition of the thermal aware workload scheduling for the distributed DC case as a constraint satisfaction problem aiming to meet the workload service level agreements and at the same time meet the heat demand of the tenants.
- Definition of a thermodynamic model to accurately estimate the heat demand needed to be generated by the IT equipment and the workload to be allocated for execution to meet the temperature setpoint defined by the tenant.
- Development of a machine learning-based model for learning and correlating the heat demand with monitored data related to the actual temperature in the room, the temperature of the heat generated by the IT equipment, and temperature setpoint.
- The heat models and workload scheduling solution were tested and validated considering the characteristics and actual monitored data from an operational distributed DC, with the results being promising in terms of meeting the heat demand and heat model’s correlation accuracy.

The rest of the paper is structured as follows: Section 2 presents the related work on DC heat reuse and workload scheduling models; Section 3 defines the thermal aware workload scheduling in a distributed DC, Section 4 describes the models defined to determine the heat demand and correlation between the workload to be executed and the temperature of the generated heat, Section 5 presents experimental results for using test data from an operation distributed DC, while Section 6 concludes the paper.

## 2. Related Work

Most approaches in the literature address the heat reuse of the common type of DC in which all the IT equipment is hosted in one building (i.e., a server room) and features a support infrastructure for power and cooling management [6,14,15]. Several heat reuse options are proposed such as for district heating, [7,8,9] hot water grid [16,17], or nearby office buildings [4,5,18]. The heat reuse policy and infrastructure are well established in Nordic European countries [6,9,10], thus the DC potential for heat reuse is usually analyzed considering this use case. The efficient heat reuse will provide new revenue streams for DCs but at the same time, several research challenges still need to be faced such as the low-grade waste heat generated by the servers, especially in the case of the air-cooled DCs and the high investment costs [1,19]. These investments usually address the deployment and installation of heat pumps that are used for raising the quality of the heat [20,21,22]. District heating is seen as one of the most promising alternatives for residual heat recycling. The DCs’ waste heat has the potential of replacing natural gas-based heat, bringing considerable cost savings and a lower carbon footprint to local communities [19,23]. 

In the area of DCs’ heat reuse, two main research topics have a strict relation to this paper’s objective and contributions: modeling and simulating the thermal characteristics of the DC and the thermal aware workload scheduling.

The first topic addresses the development of models to study the thermodynamic processes inside a DC and to determine the heat generation, transfer, and reuse characteristics [4,24,25,26]. The thermodynamics impose limits on both the maximum allowable temperature of the microprocessors and the coefficient of performance of the heat pumps [27,28]. Setting higher temperature setpoints in the server room is proposed and used to improve the quality of the recovered heat [4,29]. In this case, accurate thermal models of the server room are developed to predict the temperature variations, detect the formation of hot spots which may lead to equipment malfunctioning, and evaluate alternatives in cooling system configurations [3,11]. Computational Fluid Dynamics (CFD) models of the server room are used to run simulations for studying the interactions between servers and cooling units and their effect on the heat and temperature distribution [5,13,30]. The server heat generation and dissipation rates are analyzed and used to set the recommended temperature values for inlet air into the server room [15,31]. The CFD models of the IT server room are used to analyze the supply air temperature of the cooling system units and the inlet air temperature to find the allowed range of temperature for not damaging the equipment and activating the cooling systems [11]. As a result, higher temperature setpoints can be used for short periods while techniques such as pre- or post-cooling may be used for thermal profile adaptation [4,32]. Even though the CFD simulations provide accurate temperature predictions, they are computationally expensive [33]. Tradeoffs should be done among the accuracy of the simulation, execution time, and resource overheads. Mathematical models and machine-leaning-based approaches are used to address such tradeoffs [4,29,34] with varying levels of success. Mathematical models of the temperature evolution in a server room are presented in [35,36] addressing the thermal behavior concerning heat generation, circulation, and air-cooling system using Navier–Stokes equations expressing thermal laws or by using fast approximate solvers [37,38]. 

The combination of thermodynamics processes simulations with machine learning techniques offers promising results for determining a set of parameters empirically from monitored data [5,39,40]. In [39], a thermal forecasting model is defined and used for predicting temperatures surrounding servers in data centers. Continuous streams of temperature and airflow measurements are collected for obtaining online predictions with real-time sensor measurements. In [40], a fast converging solution is proposed using both a feed-forward network and a dynamic recurrent artificial neural network. The neural networks learn incrementally, using the incoming stream of data samples. Adaptiveness is presented as an essential feature, as the model can learn the characteristics of a server room with minimal training, and then it may continuously adapt to new data fed without retraining [41]. In [5], a heat reuse model is defined which combines the simulation of the thermodynamic processes in a server room with deep learning processes. Multi-Layer Perceptron neural networks are used for predicting the hot air temperature distribution in the server room. Some models use a set of parameters from the server room that are relevant for the thermodynamics processes and use machine learning to predict their evolution. Gradient boosting decision trees, artificial neural networks, or deep learning models are used to predict the server room temperature [42,43]. Finally, Grammatical Evolution techniques [44] and Environmentally Opportunistic Computing [45] are used for analyzing server and inlet air temperatures and predicting the temperatures, in conjunction with thermal models of DCs. The models should reflect the physical nature of the system, rather than fitting the data purely mathematically. This is enforced using rules for a model’s generation expressed grammars written in Backus–Naur form [46]. 

Thermal aware workload scheduling algorithms for heat reuse are derived from scheduling algorithms developed to minimize the cooling system energy consumption [12,47]. The main goal of these scheduling algorithms is to distribute the workload in a data center to maintain a low ambient temperature and avoid hotspot formation [47,48]. In the case of heat reuse, the workload scheduling aims to increase the efficiency of heat pump operation and to meet the heat demand of the district heating network [49]. They rely on an optimization problem, defined either reactively or proactively, whose complexity is highly dependent on the representation of the thermodynamics processes and the correlations considered among workload and power and heat demand [50,51]. Workload placement strategies considered are based on zones discretization, minimize the heat recirculation, and prioritize the servers for task allocation by observing hot airflow within the DC [47,48,51]. Scheduling methodologies common in DCs such as first-come-first-serve or backfilling do not usually consider the thermal perspective [52,53]. Machine learning-based models are proposed to infer scheduling policies with thermal features. Server room temperature prediction approaches using machine learning are proposed in conjunction with scheduling algorithms to avoid thermal stress and hotspot formation [54]. In [4], thermal aware workload scheduling is proposed to adapt the DC heat generation to the district heating demand and maximize the waste heat reuse. Neural networks are used to learn the heat generation and heat distribution in the server room. Thermal aware scheduling may consider different heuristics such as tasks and servers’ classification in hot or cold thermal prediction models, node ranking based on heat generation features, etc. [48,51,55]. In [56], an optimization problem is proposed for thermal scheduling considering the optimal setpoints for the workload distribution and the temperature in the server room. Heat flow models are proposed for determining temperatures in case of a thermal aware workload scheduling policy, while a heat recirculation matrix is used to define the thermal influences between the servers [57,58]. Algorithms are prosed to allocate workload on multiprocessors while minimizing the makespan and temperature constraints [59,60]. They aim to reduce the chip temperature while meeting the workload SLA, with the optimization problem being usually modeled as a mixed-integer linear program. Thermal aware task scheduling approaches to adjust CPU frequency based on Dynamic Voltage Frequency Scaling [61] are used to manage the energy demand and heat generation [62,63,64]. In [65], the authors build a steady and dynamical thermal interaction model of the DC. Based on these models, a task assignment and frequency optimization are performed in the first optimization stage, while the second stage uses a model predictive control (MPC) to represent the optimization problems that aim to minimize the cooling system power demand. In [12], a thermal aware consolidation mechanism is defined using a heat recirculation matrix and a set of bio-inspired algorithms that minimize overall DC energy consumption. Finally, in [56], the scheduling optimization problem is defined by considering the energy footprint reduction with thermal exchanges while incorporating both temperature and workload constraints. 

After analyzing the existing state of the art, we did not find any relevant literature approach that addresses the thermal aware workload scheduling in the case of distributed DCs while considering the IT equipment as a primary source of heat. Several papers advocate the Data Furnace as the method of heating residential homes by deploying IT equipment in their premises [66,67]. Nevertheless, they are at the stage of ideas promoting some of its advantages such as a smaller carbon footprint or a reduced total cost of ownership per server without offering an actual scheduling solution. 

In our paper, we address the identified knowledge gap in the literature by proposing a thermal-aware workload scheduling solution for distributed DCs. Our approach can consider both the service level agreements constraints of the workload to be executed and the heat demand of the residential home’s tenants. To accurately estimate the heat to be generated by the IT equipment for meeting the heat demand levels, we define thermodynamic and machine learning models. They evaluate the heat demand based on the temperature of the heat generated by the IT equipment to determine the workload to be allocated for execution such that the temperature setpoint defined by the tenant is meet. The scheduling algorithm and heat models are evaluated considering relevant data sets from an operational distributed DC showing promising results.

## 3. Thermal Aware Workload Scheduling

The distributed DC should be modeled as a collection of N isolated IT equipment (i.e., servers or micro data centers) deployed in residential buildings. Each one uses the building internet network and is deployed in a room that needs to be heated:

(1) $$DC_{Distributed}=\left\{<room_{k},server_{k}>\right\},k=1,\ldots N.$$

The IT equipment offers a direct heat source, converting the electrical energy consumed for workload execution into thermal energy (i.e., a computing heater). The thermal energy is dissipated through radiators in the surrounding air, heating the room as conventional heaters to maintain the thermal comfort of the inhabitants. The objective, in this case, is to schedule the workload tasks to be executed by the IT equipment deployed in the room k to bring and maintain the ambient temperature $T_{Room}^{k}$ close to a set point $T_{set-point}^{k}$ temperature desired by the dwellers:

(2) $$room_{k}:<T_{Room}^{k},T_{set-point}^{k}>.$$

The workload scheduling should consider the dockerized tasks and Service Level Agreements constraints in terms of computational resources to be allocated and execution time deadline. Also, the number of task migration should be kept as low as possible to minimize its impact on task SLA.

We defined the workload to be scheduled for execution over an interval [0…∇] as a set of *M* tasks:

(3) $$Workload=\left\{Task_{j},j=1,\ldots M\right\}.$$

Each task specifies the computational resources that need to be allocated, estimated execution time, and deadline according to the Service Level Agreements:

(4) $$Task:<CPU,RAM,HDD,t_{execution},t_{deadline}>,\;\;\;t_{execution},t_{deadline}\in \left[0\ldots \nabla \right].$$

The rooms should be heated by reusing residual heat generated by the servers. For each server, the computational resources, the idle, and maximum power consumption are specified:

(5) $$Server:<CPU,RAM,HDD,P_{IDLE},P_{MAX}>.$$

To allocate tasks on servers to be executed during the interval [0…∇], a scheduling matrix $W_{scheduling}$ is defined. The matrix stores the starting time for tasks execution and their allocation on specific servers:

(6) $$W_{scheduling}\in R^{N\times M},W_{scheduling}\left[Server_{k}\right]\left[Task_{j}\right]=t_{start}^{kj}.$$

If there is a task *j* scheduled to the executed-on server *k*, then:

(7) $$0<t_{start}^{kj}<T$$

otherwise, the $t_{start}^{kj}=0$.

The subset of tasks $W_{alocation}^{k}$ scheduled to be executed to each server k, from the N locations where the DC IT equipment is distributed, is determined using the task scheduling matrix:

(8) $$W_{alocation}^{k}=\{Task_{j}|W_{scheduling}\left(k\right)\left(j\right)>0,j\in \left\{1\ldots M\right\},k\in \left\{1\ldots N\right\}\}.$$

In the process of determining the scheduling matrix, several constraints need to be met. The first one is referring to the execution time of a scheduled task that is not allowed to exceed the specified execution deadline:

(9) $$t_{start}^{kj}+t_{execution}^{j}\leq t_{deadline}^{j}.$$

The second set of constraints are referring to the relation between the total computational resources requested by the tasks scheduled for execution on a server and the server’s available resources. The computational resources allocated to the tasks scheduled on a server k should be less than the total resources of that server:

(10) $$\forall t\in \left[0\ldots \nabla \right],CPU_{k}\left(t\right)\geq \sum_{j=1t_{start}^{kj}\leq t\leq t_{start}^{kj}+t_{execution}^{j}}^{M}CPU_{j}\left(t\right)$$

(11) $$\forall t\in \left[0\ldots \nabla \right],RAM_{k}\left(t\right)\geq \sum_{j=1t_{start}^{kj}\leq t\leq t_{start}^{kj}+t_{execution}^{j}}^{M}RAM_{j}\left(t\right)$$

(12) $$\forall t\in \left[0\ldots \nabla \right],HDD_{k}\left(t\right)\geq \sum_{j=1t_{start}^{kj}\leq t\leq t_{start}^{kj}+t_{execution}^{j}}^{M}HDD_{j}\left(t\right)\;.$$

Considering the resources allocated to tasks, the computational resources utilization levels for a server *k* are computed as:

(13) $$\mu_{server}^{k}\left(t\right)=\left\{\mu_{CPU}^{k}\left(t\right),\mu_{RAM}^{k}\left(t\right),\mu_{HDD}^{k}\left(t\right)\right\}$$

(14) $$\mu_{CPU}^{k}\left(t\right)=\frac{\sum_{j=1t_{start}^{kj}\leq t\leq t_{start}^{kj}+t_{execution}^{j}}^{M}CPU_{j}\left(t\right)}{CPU_{k}\left(t\right)}$$

(15) $$\mu_{RAM}^{k}\left(t\right)=\frac{\sum_{j=1t_{start}^{kj}\leq t\leq t_{start}^{kj}+t_{execution}^{j}}^{M}RAM_{j}\left(t\right)}{RAM_{k}\left(t\right)}$$

(16) $$\mu_{HDD}^{k}\left(t\right)=\frac{\sum_{j=1t_{start}^{kj}\leq t\leq t_{start}^{kj}+t_{execution}^{j}}^{M}HDD_{j}\left(t\right)}{HDD_{k}\left(t\right)}\;.$$

The power consumed by the server is determined using the utilization ratio and the power characteristics of the servers as:

(17) $$P_{server}^{k}\left(t\right)=f_{power}\left(\mu_{CPU}^{k}\left(t\right),\mu_{RAM}^{k}\left(t\right),\mu_{HDD}^{k}\left(t\right),P_{idle}^{k},P_{MAX}^{k}\right).$$

For the function $f_{power},$ we used a linear model to compute the power considering the idle and maximum power of the server k and the CPU utilization level [68]:

(18) $$P_{server}^{k}\left(t\right)=P_{idle}^{k}+\mu_{CPU}^{k}\left(t\right)*\left(P_{MAX}^{k}-P_{idle}^{k}\right).$$

The energy consumed by the $server_{k}$ over the time interval [0…∇] can be computed as an integral of the power over this time window:

(19) $$E_{server}^{k}=\int_{0}^{\nabla }P_{server}^{k}\left(t\right)dt\;.$$

Among the computational resources considered for a server, the main heat source is the CPU, which is responsible for 30% up to 65% of the total heat dissipated and also has the highest temperature. Other resources have less impact with varying proportions, thus we denoted them with parameters that can be determined empirically. According to the law of energy conservation, the electrical energy consumed by the servers can be transformed into heat that is dissipated in the room where the servers reside over the interval [0…∇]. This heat can be split according to the nc computational resources of the server, with each having been assigned a weight ω according to the proportion of the thermal energy generated:

(20) $$Q_{server}^{k}\left(\nabla \right)=\sum_{i=1}^{nc}\omega \left(i\right)*E_{component}^{k}\left(i\right)\;.$$

Considering the tasks allocated over the interval [0…∇], the function $f_{schedule}:R^{M}\to R^{nc}$ estimates for each server, the energy consumed by each component, which can be used to estimate the server heat generation.

(21) $$E_{component}^{k}=f_{schedule}\left(W_{alocation}^{k}\right)$$

The heat dissipation in each room leads to a temperature modification according to a function $f_{Q}$ that estimates the ambient temperature $T_{Room}^{k}$ modification over the interval [0…∇]:

(22) $$T_{Room}^{k}\left(\nabla \right)=f_{Q}^{k}\left(T_{Room}^{k}\left(0\right),Q_{server}^{k}\left(\nabla \right)\right).$$

In our case, the workload scheduling for a distributed DC aims to allocate the workload tasks on the IT servers to optimally generate the heat according to the resident’s demand. The room temperature setpoints need to be reached as fast as possible and are kept constant over the rest of the time interval, with no task migrations, in each of the N locations where the IT equipment is distributed.

The thermal aware workload scheduling problem is modeled as a constraint satisfaction problem to determine the optimal workload scheduling matrix $W_{scheduling}$ such that the set-points temperatures in each of the N rooms are met (see Algorithm 1). Its solving process involves nonlinear programming [69] because of the non-linearities of the objective function and the workload scheduling function $f_{schedule}$ and the continuous values of the $W_{scheduling}$ matrix. Also, it is an NP-hard problem [70], thus an approximation algorithm is needed to determine a solution.
**Algorithm 1** Thermal aware workload scheduling**Input:**$DC_{Distributed}$, N the number of distributed servers, M the number of workload tasks, $T_{Room}$ the temperatures in the rooms where the servers are distributed, $T_{set-point}$ the desired temperature in the rooms. **Output**: $W_{scheduling}$ matrix keeping the tasks scheduling on servers $\;\;\;\;\;\;\;\;\;\;\;\;\;\;\;\;\;OptimizationGoal:determineW_{scheduling}toMIN\left(\sum_{k=1}^{N}distance\left(T_{Room}^{k},T_{set-point}^{k}\right)\right)$
Considering the following constraints for each server k=1…N

$C1:W_{alocation}^{k}=\{Task_{j}|j\in \left\{1\ldots M\right\}andW_{scheduling}\left(k\right)\left(j\right)>0\}$

$C2:Q_{generated}^{k}\left(T\right)=\sum_{i=1}^{nc}\omega \left(i\right)*f_{schedule}\left(W_{alocation}^{k}\right)$
$C3:T_{Room}^{k}\left(\nabla \right)=f_{Q}^{k}\left(T_{Room}^{k}\left(0\right),Q_{server}^{k}\left(\nabla \right)\right)$

$C4:t_{start}^{kj}+t_{execution}^{j}\leq t_{deadline}^{j}$

$C5:\forall t\in \left[0\ldots T\right],CPU_{k}\left(t\right)\geq \sum_{j=1t_{start}^{kj}\leq t\leq t_{start}^{kj}+t_{execution}^{j}}^{M}CPU_{j}\left(t\right)$

$C6:\forall t\in \left[0\ldots T\right],RAM_{k}\left(t\right)\geq \sum_{j=1t_{start}^{kj}\leq t\leq t_{start}^{kj}+t_{execution}^{j}}^{M}RAM_{j}\left(t\right)$

$C7:\forall t\in \left[0\ldots T\right],HDD_{k}\left(t\right)\geq \sum_{j=1t_{start}^{kj}\leq t\leq t_{start}^{kj}+t_{execution}^{j}}^{M}HDD_{j}\left(t\right)$

**Figure 2:**
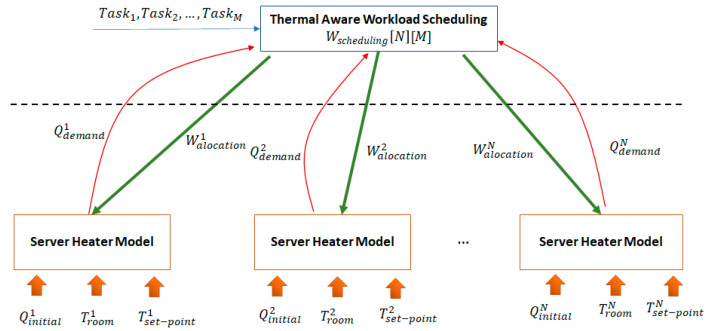
Thermal aware workload scheduling optimization subproblems.

The main challenge is the unknown nature of the $f_{Q}^{k}$ function that may feature complex representations, making the utilization of optimization algorithms derived from stochastic gradient descent unfeasible [71]. To address this, we split the thermal aware scheduling problem into two subproblems (see Figure 2). For the first subproblem involving estimates for each room k, the heat that needs to be generated by the server to make the transition from $T_{Room}^{k}\left(0\right)$ to $T_{set-point}^{k}$. The second one determines the workload scheduling matrix $W_{scheduling}$ such that a large enough workload is scheduled for execution on each server to generate heat to meet the demand. The latter problem can be solved using an adaptation of the Multiple Knapsack Problem [72].

In the case of the first subproblem, we aimed to approximate the heat demand $Q_{Demand}^{k}$ needed over the interval [0…∇] to make the transition from $T_{Room}^{k}\left(0\right)$ to $T_{set-point}^{k}$ and then keep the temperature constant at the set point level. It can be solved by defining a function $f_{Q}^{-1}$ that estimates the amount of heat needed to make the temperature transition:

(23) $$Q_{demand}^{k}\left(\nabla \right)=f_{Q}^{-1}\left(T_{Room}^{k}\left(0\right),Q_{initial}^{k},T_{set-point}^{k}\right).$$

In the case of the second subproblem, the goal is now to determine the workload scheduling matrix $W_{Scheduling}$ so that the heat generated by each server matches closely the heat demand of the corresponding room:

(24) $$MIN(\sum_{k=1}^{N}distance\left(Q_{demand}^{k},Q_{generated}^{k}\right))\;.$$

The optimization still involves nonlinear programming, but the functions are not defined as black-box models. As result, it can be solved using approximation algorithms used to determine solutions for the Multiple Knapsack Problem [72]. Using this time discretization over the interval [0…∇], the scheduling problem can be reduced to an N × ∇ knapsack problem, where the N × ∇ knapsacks volumes correspond to the values of the heat demand to be generated on each of the ∇ intervals in each of the N rooms, while the items being packed are the workload tasks to be deployed on servers and executed.

## 4. Heat Demand Estimation

The thermal aware workload scheduling model described in the previous section needs accurate estimations of the heat demand to be generated by the IT equipment such that the temperature set point in the room set by the residents is met. In this section, we propose two models for implementing the $f_{Q}^{-1}$ function used to determine the head demand estimations.

### 4.1. Server Heat Transfer Model

We defined a thermodynamic model of the server calibrated with measurements. To ease the representation, we assumed that the room temperature $T_{Room}^{k}$ is constant during the workload scheduling period [0…∇]. This means that all the power generated by the server $Q_{server}$ is dissipated in the surrounding environment by rising to the ceiling, while cold air from the floor passes through the computing element radiator, leading to an energy loss $Q_{loss}$ of the room. 

The server power consumption for executing the allocated tasks, the temperature of the heat generated ($T_{server}$), and room temperature are linked as follows [73]:

(25) $$P_{server}=Q_{server}=c_{server}\times \frac{∆T_{server}}{∆t}+f_{air}\times c_{a}\times \varepsilon_{server}\times \left(T_{server}-T_{Room}\right)$$

where $c_{server}$ is the server heat capacity, $f_{air}$ represents the airflow over the server surface, $c_{air}$ is the specific heat capacity of air, and $\varepsilon_{server}$ is the thermal server efficiency. Thermal efficiency is defined as the ratio of real to maximum power transfer between the server’s body and the airflow and can be experimentally determined by measuring the temperature of emerging airflow denoted as $T_{ex}$.

(26) $$\varepsilon_{server}=\frac{P_{server}}{P_{server}^{max}}=\frac{f_{air}\times c_{air}\times \left(T_{ex}-T_{ROOM}\right)}{f_{air}\times c_{air\times }\left(T_{server}-T_{ROOM}\right)}=\frac{T_{ex}-T_{ROOM}}{T_{server}-T_{ROOM}}$$

The changes in the server power $P_{server}$ influence the temperature of the heat generated. We assumed that the power is changed linearly with respect to time with the ratio R:

(27) $$P_{server}\left(t\right)=R\;\times t+P_{server}\left(0\right).$$

We defined the transient state of server-generated heat temperature $T_{server}$ over the time interval in which $P_{server}$ changes, while the equilibrium state was defined to be when the $P_{server}$ reaches a constant value. Relation (25) can be used to define both the transient and the equilibrium regimes of the computational equipment. The transient temperature is defined in Equation (28) as a solution of (25), as a function of the time coordinate, while the solution in the equilibrium regime is given by Equation (29).

(28) $$T_{server-transient}=\left[A\times e^{-\frac{t}{\left(\frac{C_{server}}{f_{air}\times c_{a}\times \varepsilon_{server}}\right)}}+R\times \frac{t}{f_{air}\times c_{a}\times \varepsilon_{server}}\right]-\;-\frac{R\times C_{server}}{{\left(f_{air}\times c_{a}\times \varepsilon_{server}\right)}^{2}}+T_{Room}+\frac{P_{server\;}\left(0\right)}{f_{air}\times c_{a}\times \varepsilon_{server}}$$

(29) $$T_{server-equilibrium}=B\times e^{-\frac{t}{\left(\frac{C_{server}}{f_{air}\times c_{a}\times \varepsilon_{server}}\right)}}+T_{Room}+\frac{P_{server-\mathrm{final}}}{f_{air}\times c_{a}\times \varepsilon_{server}}$$

In Equations (28) and (29), the constants A and B are fixed by the initial conditions in each case. The equilibrium regime defined in Equation (29) tends asymptotically to a state independent of time due to the rapid exponential decay of the first term, eventually reaching a state depending only on the power workload and predicting the behavior of $T_{server}$ with respect to the change of $P_{server}$. The server temperature after the equilibrium temperature setting can be estimated with Equation (30), derived from (29) when the first term decays to zero.

(30) $$T_{server-final}\approx \;T_{ROOM}+\frac{P_{server-final}}{f_{air}\times c_{a}\times \varepsilon_{server}}$$

The parameters used in defining the server as a heater model are detailed in Table 1, mentioning which parameter should be measured, and which are tuned experimentally for a particular model. The parameters from Table 1 that are determined experimentally can be computed while considering a set of measurements from the physical server room, allowing the model to be fitted to the exact configuration of the real-world system. 

Finally, after reaching the equilibrium temperature, the assumption that $T_{Room}$ is constant can be relaxed, as the computational equipment will dissipate heat in the room, leading to a temperature change. Considering the equations developed above, the heat per unit of time demanded by the servers overtime to pass the transient regime and transition the temperature $T_{Server}$ to a temperature $T_{server-equilibrium}$ close to $T_{server-final}$ can be computed as the server demand over the transient regime:

(31) $$Q_{demand}\left(t\right)=P_{server}\left(t\right)=R\times t+P_{server}\left(0\right).$$

However, to transition the room temperature from $T_{ROOM}$ to $T_{set-point}$, we considered that the server has reached a state sufficiently close to the asymptotic equilibrium and the relation between $T_{server-final}$ and $T_{ROOM}$ is approximately linear, so for small changes and fluctuations, the difference between the two temperatures is largely unchanged. Using simple thermodynamic considerations once again, it can then be stated that:

(32) $$(P_{server-final}-P_{loss})∆t=M\times c_{a}\times \left(T_{set-point}-T_{ROOM}\right)$$

which is an expression of the energy required to heat the room from $T_{ROOM}$ to $T_{set-point}$ in time ∆t after the server has reached its equilibrium temperature, M is the mass of the air in the room, computed by multiplying the room volume V by the air density $\rho_{air},$ and $P_{loss}$ is the energy lost per unit time by the room due to air drafts and imperfect thermal insulation. Thus, from (32) we can deduce the expression:

(33) $$P_{server-final}=P_{loss}+\frac{M\times c_{a}\times \left(T_{set-point}-T_{ROOM}\right)\;}{∆t}.$$

We can now obtain the more useful expression for $Q_{Demand}\left(t\right)$, given $T_{set-point}$ for the air in the room, knowing that $Q_{Demand}\left(\nabla \right)=P_{server-final}$ and that the increase in power is linear:

(34) $$Q_{Demand}\left(t\right)=\frac{P_{loss}+\frac{M\times c_{a}\times \left(T_{set-point}-T_{ROOM}\right)\;}{∆t}-P_{server}\left(0\right)}{\nabla }\times t+P_{server}\left(0\right),\forall t\in \left[0\ldots \nabla \right]$$

It should be noted that the time in which the room heats up, ∆t, is a parameter that can be chosen freely, and should not be made too short, as this will cause the server to overheat i.e., exceed the recommended functioning temperature of approximately 35 °C. The limiting minimum size ∆t is easily deducible from previous expressions. Finally, if the room is to be heated, $P_{server-final}$ must be larger than $P_{loss}$, otherwise, the room will not receive any net heat and will not increase its temperature.

### 4.2. Machine Learning-Based Model

**Figure 3:**
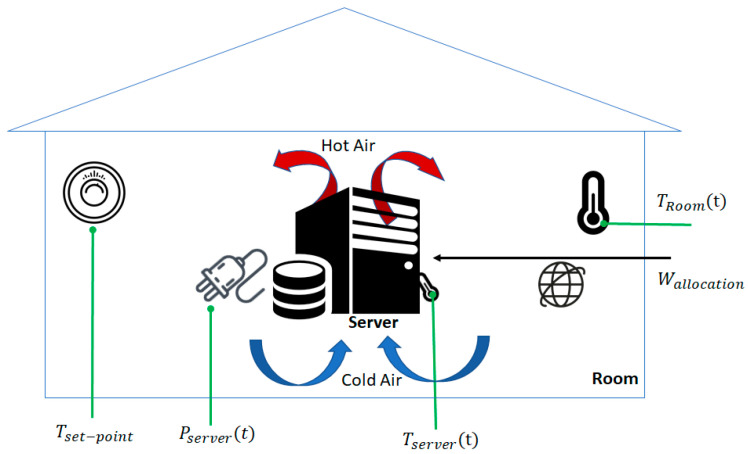
Measurements used in the machine learning processes.

The machine learning model aims to infer and correlate the heat demand that needs to be generated by the server executing the allocated workload out of monitored data (see Figure 3). We considered that the room temperature is collected using heat sensors placed at a certain distance from the server. The heatsink temperature is collected using the server’s embedded temperature sensor, whereas the power consumption is obtained using a wattmeter.

The machine learning model takes as inputs the actual room temperature $T_{Room}\left(0\right)$, the initial power consumption $P_{server}\left(0\right)$, the desired room temperature, $T_{set-point}$, and provides as output the heat demand that the server should generate by executing workload, to reach the desired temperature, $T_{Room}\left(\nabla \right).$

All data needs to be smoothed so that outliers are eliminated. We aimed to detect areas of interest, defined by a high correlation coefficient between the heatsink and ambient temperature, limited between two local peaks of the temperatures and power. Thus, we used the Pearson Correlation Coefficient, as defined in Equation (35), to measure the linear correlation between two datasets. Only relevant samples with values larger than a threshold were considered for further processing, namely data where the room temperature, server temperature, and power consumed by the server present similar patterns.

(35) $$r_{T_{heatsink},T_{ROOM}}^{window}=\frac{cov\left(T_{server},T_{ROOM}\right)}{\sigma_{T_{server}}\sigma_{T_{ROOM}}}>threshold$$

As the area of interest still may contain data that might be irrelevant for our final purpose, it needed further processing. Only the initial and final temperatures were selected from the scheduling interval [0…∇]. They represent the relevant information for predicting the power consumption, given the temperature. The initial temperatures can be computed as the mean temperature before a sudden change, whereas the final ones can be computed as the mean temperature after stabilization. The same goes for power, consequently obtaining the four needed values:

(36) $$\left[\begin{array}{ccc} <T_{Room}\left(t_{1}\right),P_{server}\left(t_{1}\right),T_{server}\left(t_{1}\right)> & \to & P_{server}\left(t_{1}+\nabla \right)\\ <T_{Room}\left(t_{2}\right),P_{server}\left(t_{2}\right),T_{server}\left(t_{2}\right)> & \to & P_{server}\left(t_{2}+\nabla \right)\\ \ldots & \ldots & \ldots \\ <T_{Room}\left(t_{n}\right),P_{server}\left(t_{n}\right),T_{server}\left(t_{n}\right)> & \to & P_{server}\left(t_{n}+\nabla \right) \end{array}\right]$$

where $t_{1},t_{2},\ldots t_{n}\in \left[0\ldots \nabla \right]$, are timestamps of the data acquisition.

Several models were implemented and used to learn the behavior of the function $f_{Q}^{-1}$ used to estimate the heat demand. Table 2 presents the description of the models and their configuration determined empirically on the test data.

## 5. Evaluation Results

**Figure 4:**
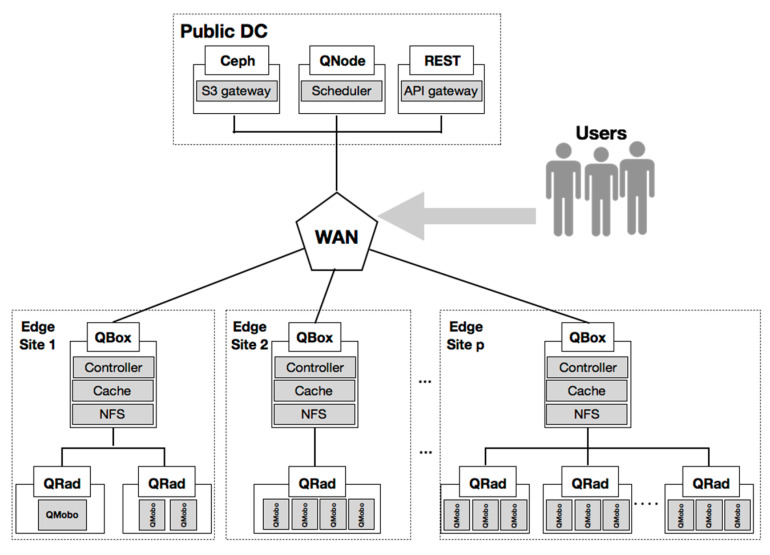
Test case distributed DC.

To evaluate the thermal aware workload scheduling and the heat demand estimation models, we considered a test case distributed DC [74] composed of a main DC and a set of edge sites (see Figure 4). Each edge site hosts a workload distribution node, the QBox, and a set of server heater nodes, the QRads, that provide heat by executing the workload provided by the QBox. The QRad is a server-heater with no moving parts and three motherboards equipped with CPUs that execute workload and dissipate the heat in the surrounding environment, leading to a temperature rise. Each QRad node consumes about 400 W of electrical energy and generates an amount of roughly 400 W of heat, depending mainly on the CPU model. The workload that runs on the QRad is composed of tasks that use full CPU resources, such as 3D animation or financial risk computing. 

A sensors-based monitoring infrastructure was used to acquire relevant data regarding the Ambiental temperature $T_{Room}$, power consumption of the QRad ($P_{server}$) and temperature of the server $\left(T_{server}\right).$ The data acquired spans over several months, being recorded at a granularity of 10 s. Both the characteristics of the QRad heaters and monitored data acquired by the installed infrastructure are the inputs of our study.

**Figure 5:**
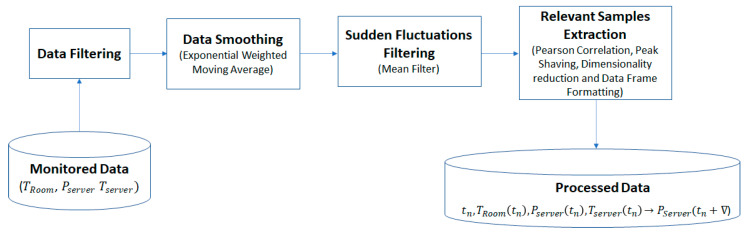
Data pre-processing pipeline.

However, the monitored data used to model the thermal behavior of the QRad heaters must be extracted for larger intervals that show suggestive temperature and power changes. Thus, a pipeline of data pre-processing operations was employed to extract the relevant data samples (see Figure 5). 

The acquired data were smoothed using an exponential weighted moving average window with the span of 90 data points corresponding to a time window of 15 min. A mean filter with a window span of 60 data points, corresponding to a time window of 10 min, was applied to ignore sudden fluctuations. To find the exact times when the ambient ($T_{Room})$ and the server temperatures ($T_{server})$ rise or fall together, Pearson’s r coefficient was used. This was applied on a rolling window with the span of 360 data points corresponding to a time interval of 1 h to compute the correlation. The samples corresponding to intervals where the scores exceeded 0.5 were considered. Finally, local maximum and minimum peaks were found over window sizes of 180 data points corresponding to 30 min time intervals. Only samples that started at a local minimum and ended at a local maximum or vice-versa were taken into consideration. The process involved manual inspection of the selected data and fine fitting so that the included regions could also contain the power instant change, which sometimes takes place before a local peak. 

**Figure 6:**
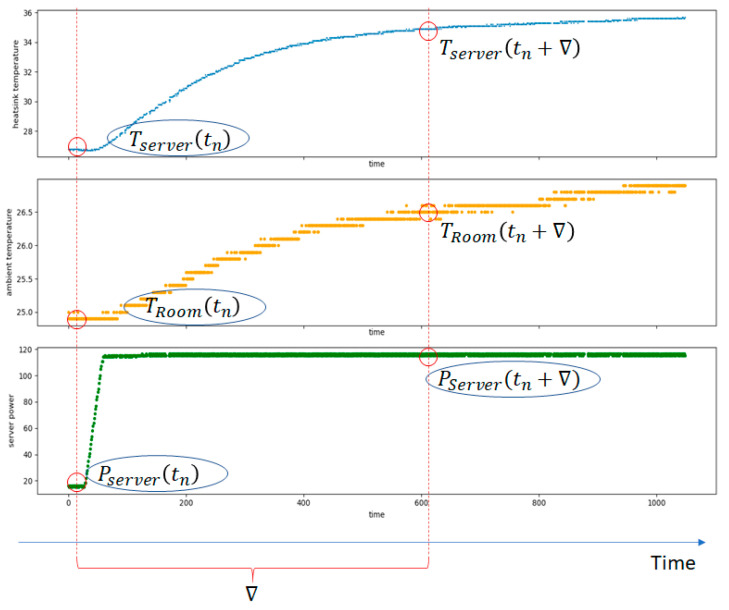
Example of relevant data samples obtained using the pre-processing pipeline.

Figure 6 shows a relevant data sample obtained by filtering, smoothing, and applying Pearson’s coefficient. The length of the time interval ∇ is determined between the start of the temperature change and the end of the temperature change. Analyzing the data set, we determined ∇ for each sequence to record most temperature changes and to reach a steady-state situation. Since most of the samples show that the server power change is similar to a linear function, only the final server power consumption was considered ($P_{Server}\left(t_{n}+\nabla \right))$. This can be computed based on the initial state parameters at the time $t_{n}$: the ambient temperature at the time $T_{Room}\left(t_{n}\right)$, the server temperature, $T_{server}\left(t_{n}\right)$, and the server power demand $P_{server}\left(t_{n}\right)$.

Firstly, we considered the server heat transfer model detailed in Section 4.1. The model was calibrated by fitting some of its parameters on the actual data gathered from the QRad heaters. The heat model parameters from Table 1 (i.e., the server heat capacity $C_{server}$, the server thermal efficiency, $\varepsilon_{server}$) have to be computed to fit best on monitored data, while the airflow over the server (i.e., $f_{air}$) has to also be estimated.

The fitting process consists of feeding a trace of processed data in the form $T_{ROOM}\left(t_{n}\right),P_{server}\left(t_{n}\right),T_{server}\left(t_{n}\right)\to P_{Server}\left(t_{n}+T\right)$ for each QRad server CPU to an optimizer that can compute the model parameters using gradient descent-based algorithms [71]. The processed data samples were split into 80% training data for model fitting, while 20% was used to validate the model. The fitting process is performed iteratively, at each step a sample of training data is read, and the variables $C_{server},\varepsilon_{server}$ and $f_{air}$ were varied from a set of initial values until they fit best the data. The sequential fitting was done using a custom script from the SciPy library, namely the *scipy.optimize* method [75], applied on each data sample, recording values of $C_{server},\varepsilon_{server}$ and $f_{air}$ from the fit process and using them as initial guesses for the next sample’s fit. Repeating this operation gave us progressively refined values of the server’s parameters. 

**Figure 7:**
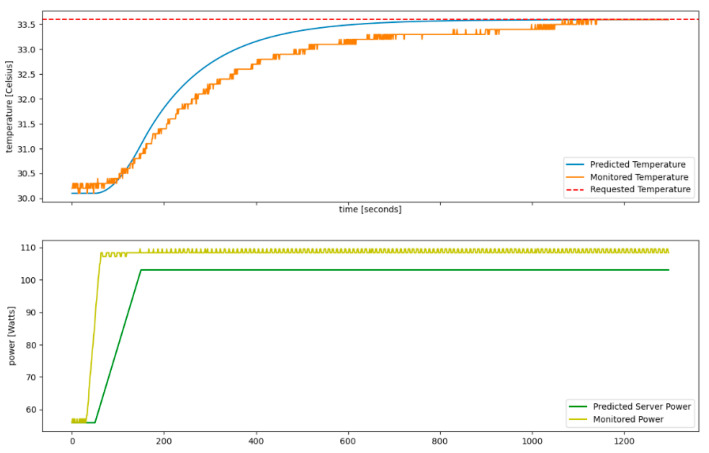
Power prediction for temperature change.

After having determined the characteristics for each QRad server, we predicted the change in power workload on the testing data. This was done by fixing $C_{server},\varepsilon_{server},$ and $f_{air}$ to the values obtained, reading $T_{server}$ and $T_{Room}$ from the data and finally obtaining our prediction for the change in $P_{server}$, which we would compare to the one in the data. A prediction chart is shown in Figure 7, illustrating as a linear function the power change for a server heater (depicted in green line) to increase the temperature (depicted in blue line) to match the requested temperature as closely as possible (depicted in red line), over a time interval of 20 min. 

The model was evaluated on a set of scenarios, with both fitting processes described above, achieving the average prediction accuracy from Table 3.

Secondly, we evaluated the machine learning-based heat model presented in Section 4.2. A set of several machine learning algorithms were implemented to model the QRad server heat generation. The Linear and Polynomial Regression, Random Forest Regression, Support Vector Regression, and K Neighbors Regression were implemented using Python’s SciKitLearn [76] library. The Multi-Layer Perceptron was implemented using Keras [77] and Tensorflow [78] while for the Gradient Boosting Regression, the XGBoost’s [79] was used.

The dataset processed using the defined pipeline of operations can be split into train and test subsets, with the proportions of 0.8 and 0.2 of the initial data. The models were validated using 5-fold cross-validation and were evaluated by computing the Root Mean Square Error (RMSE), Root Mean Percentage Square Error (RMSPE), the coefficient of determination R2 (R Squared), the error mean, the error standard deviation, and MAPE. The average results obtained are listed in Table 4.

As Table 4 shows, considering the MAPE and the RMSPE, the best results are obtained by the Gradient Boosting Regression (GBR). These are the most relevant metrics for prediction accuracy, considering the percentages of the errors. They are showing that the GBR model can predict the heater power demand with less than 5% error, corresponding to less than 10 W of power. However, by analyzing the mean and the standard deviation on the error besides the RMSPE and the MAPE, the Random Forrest Regression (RFR) gives better results, showing a small error distribution considering the mean error. Finally, the R2 metric for the two models is closest to 1, with values of 0.94 for GBR and 0.92 for RFR. Looking at all calculated metrics, we considered that the GBR gives the best results considering the datasets collected from the QRad heaters and is most suitable for being used in a thermal aware workload scheduling algorithm.

Thirdly, we evaluated the thermal aware workload scheduling solution presented in Section 3. The heating requirements for a room can be determined by several models used in the industry to determine the recommended size of the heaters [80]. The main factors that influence the required power of the heaters in a room are the volume of the room and the caloric coefficient of the room. The latter has a value between 40 and 70 $\mathrm{kcal}/m^{3}$ and is influenced by the thermal insulation of the room, the number of exterior walls, the number of windows, and their size and type. The power needed to heat a room according to industry standards can be computed as:

(37) $$P_{Room}^{Reccomended}=V_{ROOM}*C_{ROOM}^{cal}*c_{W}^{cal}$$

where $V_{ROOM}$ is the volume of the room expressed in cubic meters $m^{3}$, $C_{ROOM}^{cal}$ is the caloric coefficient of the room expressed in $\frac{\mathrm{kcal}}{m^{3}},$ and $c_{W}^{cal}$ is the conversion factor from kcal to W having an approximate value of 1.163 W/mcal.

We determined the required heating power for rooms with different configurations and we assessed the number of QRad heaters needed to be installed (see Table 5). We considered that the heat losses from the room are much smaller than the heat generation ratio.

From the electrical energy consumption perspective, a heating system based on electrical heater radiators would consume the same amount of electricity as the server heaters for generating the same amount of heat. This is a result of the server design having no moving components, thus according to the law of energy conservation, most of the electrical energy consumed by the servers is converted into thermal energy and dissipated as heat in the room. Finally, as the server design is like a standard heater, they are installed in the room in the same positions as standard heaters, requiring no ventilation system for heat recirculation.

To illustrate our case study, we have considered the power and thermal behavior of 4 QRad server heaters containing three motherboards, each with an Intel^®^ Core™ i7-6950X CPU with 10 cores [81], totaling 12 CPU with 120 cores located in a room with a volume of 30 cubic meters, that require roughly 1.8 kWh for heating. 

**Figure 8:**
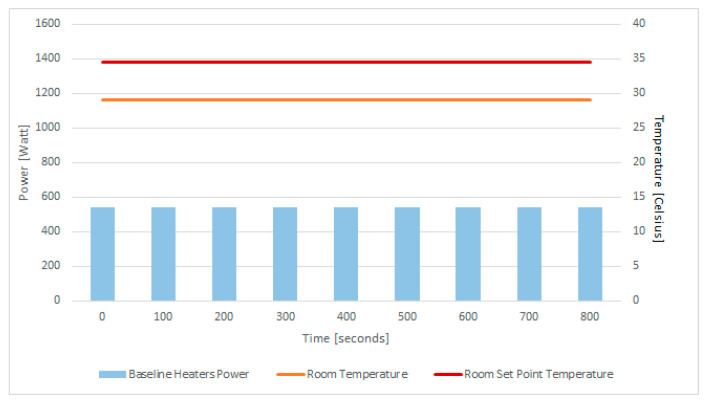
Initial conditions: the 4 QRad server heaters’ total power demand and initial temperatures.

The server’s initial temperature is 29 °C and their total power demand is 540 W, while each of the four servers only has one of the three CPUs active with one core active out of the 10 total cores. The temperature and power status of the server over a time interval of 15 min is displayed in Figure 8, showing a steady-state condition in the room. The desired server temperature is 34.5 degrees Celsius, requiring the heater to generate thermal energy to increase the room temperature.

**Figure 9:**
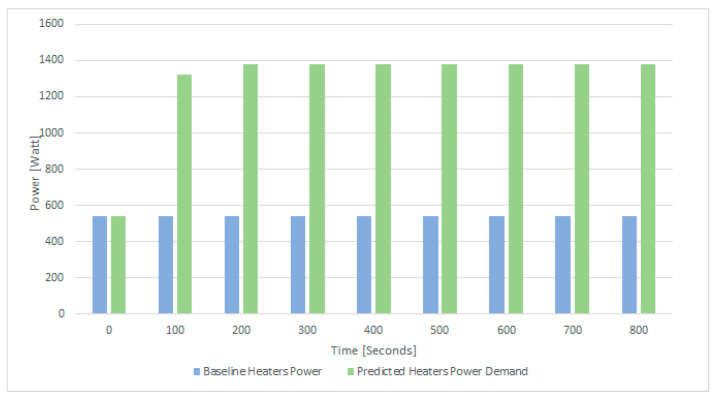
GBR heat demand estimation model power prediction.

Figure 9 shows the power demand prediction using the GBR model for total heat demand estimation for the 4 QRad server heaters to transition the room temperature to the set-point temperature. Each of the 12 CPUs’ estimated power demand is roughly 115 W, meaning that the 4 QRads will generate approximately 1.4 kW of heat.

**Figure 10:**
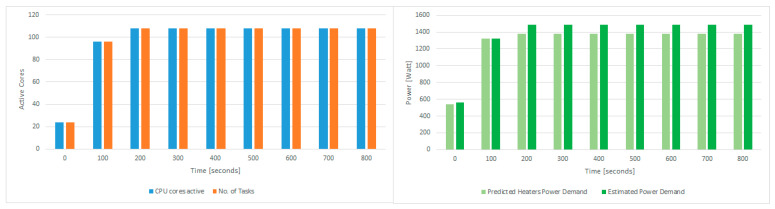
CPU active cored due to task scheduling on the **left** and CPU power demand estimated and predicted on the **right**.

Based on the power demand given by the GBR model for heat demand estimation, the thermal aware workload scheduling algorithm aims to schedule workload for execution so that the CPU usage leads to the required power demand. We considered a set of synthetic tasks running in Docker, each requiring 1 active CPU core processing near 100% and 1 GB of RAM. The task allocation result after solving the optimization problem from Section 3 is shown in Figure 10 left. For each of the 4 servers and their 3 CPUs, the plan activates 8 cores to run at a time interval of 0–100 s and 9 cores to run in the time interval of 100–800 s. Thus, in total 108 cores will be active during the heating period, leading to a total of 1488 W, shown in Figure 10-right, a value close to the predicted power demand of 1400 W estimated by the GBR model.

**Figure 11:**
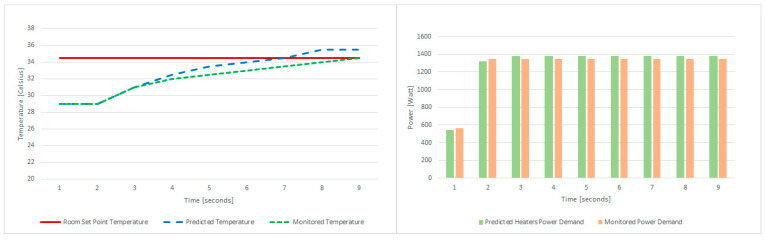
QRad Simulation Results: temperature evolution (**left**) and power demand evolution (**right**).

Finally, we use the data logs to estimate the thermal behavior of the 4 servers for the load computed by the thermal aware scheduling algorithm while considering the predicted load of the GBR model for heat demand estimation. As Figure 11-right shows, the server power demand is close to the predicted heat demand, being able to match it with 97% accuracy. As a result of the power demand and workload execution, the servers dissipate heat in the room, leading to the temperature evolution from Figure 11-left. The blue dotted line shows the room temperature evolution because of simulating the installed QRad behavior with the power demand depicted in Figure 11-right. The green dotted line depicts the room monitored temperature extracted from the data logs, showing a temperature increase close to the simulated QRad behavior and matching the set-point temperature after 800 s.

## 6. Conclusions

In this paper, we consider the case of distributed DCs and associated problems related to heat reuse when the servers are installed in residential homes are used as a primary source of heat. We propose a workload scheduling solution based on constraint satisfaction to allocate workload on severs for reaching and maintaining the desired temperature set-point in residential homes by reusing their residual heat. Two models were defined to correlate the heat demand with the amount of workload to be executed by the servers: a mathematical model derived from thermodynamic laws calibrated with monitored data and a machine learning model able to predict the amount of workload to be executed by a server to reach a desired temperature set point. The results obtained considering monitored data from an operation distributed DC are promising. The workload scheduling solution can distribute the workload so that the temperature setpoints are meet in a reasonable time, while the server heat and power demand correlation models achieve good accuracy levels.

## Figures and Tables

**Table 1:** Server heater model parameters.

Parameter	Description	Parameter Type
$c_{air}$	Air specific heat capacity, defined as a physical constant for air property dependent on temperature	Constant
$c_{server}$	Server heat capacity, defined as a physical property of the server, intrinsic to the system	Experimentally determined
$f_{air}$	Airflow of air pumped by the cooling system over the servers, measured in $\frac{m^{3}}{s}$, the intrinsic parameter of the system.	Measured during cooling system operation
$\varepsilon_{server}$	Server thermal efficiency, defined by Equation (22), considered an intrinsic parameter of the system	Experimentally determined
$T_{ROOM}$	Room temperature measured by sensors during system operation	Measured
$T_{server}$	Server temperature measured by sensors, directly influencing the room temperature	Measured
$P_{server}$	Server power demand due to workload execution	The output of the model

**Table 2:** Machine learning models used for determining the heat demand.

Model Type	Model Description
Linear Regression	The basic linear regressor was used to determine the baseline for prediction accuracy
Polynomial Regression	A second-degree polynomial regressor. Multiple degrees were considered, but the validation score began to drop after the degree was set to 2.
Gradient Boosted Regression	90 estimators with a maximum depth of 4. The samples had a minimum split of 5 and the learning rate was 0.1. The loss was computed using the least-squares method.
Random Forest Regression	9 estimators with a maximum depth of 4 are defined.
Support Vector Regression	A support vector regressor with kernel type of radial basis function and parameters: C=100,γ=0.01,ε=0.1
K Neighbors Regression	The K-Nearest Neighbors Regression with 2 neighbors and uniform weights.
Deep Learning Regression	Multi-Layer Perceptron having one input layer, two hidden layers of 128 and 256 neurons, and one output layer. The activation function for the hidden layers is of type Rectified Linear Units (ReLU), and 500 epochs were used for training. The loss function was the mean squared error and the optimizer ADAM. Early stopping was employed with the patience of 50 epochs and a minimum validation loss as the monitor.

**Table 3:** Evaluation of the server heat transfer model power prediction capability.

Server	MAPE	RMSPE	RMSE
QRad Heater	11.98	14.28	20.15

**Table 4:** Evaluation results of machine learning-based heat models.

Model	RMSE	R2	Error Mean	Error Standard Deviation	RMSPE	MAPE
Linear Regressor	14.4	0.89	−0.66	14.04	8.62	13.36
Polynomial Regression	33.12	0.39	2.05	29.19	23.38	13.65
Random Forest Regressor	10.04	0.92	−2.06	9.55	7.6	5.06
Gradient Boosting Regressor	10.65	0.94	−1.12	10.32	7.14	4.74
Support Vector Regression	16.28	0.85	2.33	15.02	9.71	6.89
K Neighbors Regressor	13.7	0.84	1.09	12.85	10.54	6.27
Multi-Layer Perceptron Deep Neural Network	33.09	1	1.92	24.54	20.62	17.29

**Table 5:** Residential heating equipment sizing case study.

$V_{ROOM}\left[m^{3}\right]$	Number of Exterior Walls	Thermal Insulation	$C_{ROOM}^{cal}$ $\;[\mathrm{kcal}/m^{3}]$	$P_{Room}^{Reccomended}\;[\mathrm{Watt}]$	Number of QRad Heaters
30	1	Yes	51	1838	4
	1	No	55	1991	4
	2	Yes	55	1991	4
	2	No	60	2144	4
35	1	Yes	51	2100	4
	1	No	55	2275	4
	2	Yes	55	2275	4
	2	No	60	2450	5
50	1	Yes	51	3000	6
	1	No	55	3250	6
	2	Yes	55	3250	6
	2	No	60	3500	7

## Data Availability

Data sharing is not applicable to this article.

## References

[1] Pärssinen M., Wahlroos M., Manner J., Syri S. (2019). Waste heat from data centers: An investment analysis. Sustain. Cities Soc..

[2] Bashroush R. Data Center Energy Use Goes Up and Up and Up, January 2020, Uptime Institute. https://journal.uptimeinstitute.com/data-center-energy-use-goes-up-and-up/.

[3] Capozzoli A., Primiceri G. (2015). Cooling Systems in Data Centers: State of Art and Emerging Technologies. Energy Procedia.

[4] Antal M., Cioara T., Anghel I., Pop C., Salomie I. (2018). Transforming Data Centers in Active Thermal Energy Players in Nearby Neighborhoods. Sustainability.

[5] Antal M., Cioara T., Anghel I., Gorzenski R., Januszewski R., Oleksiak A., Piatek W., Pop C., Salomie I., Szeliga W. (2019). Reuse of Data Center Waste Heat in Nearby Neighborhoods: A Neural Networks-Based Prediction Model. Energies.

[6] Wahlroos M., Pärssinen M., Rinne S., Syri S., Manner J. (2018). Future views on waste heat utilization—Case of data centers in Northern Europe. Renew. Sustain. Energy Rev..

[7] Nielsen S., Hansen K., Lund R., Moreno D. (2020). Unconventional Excess Heat Sources for District Heating in a National Energy System Context. Energies.

[8] Wahlroos M., Pärssinen M., Manner J., Syri S. (2017). Utilizing data center waste heat in district heating—Impacts on energy efficiency and prospects for low-temperature district heating networks. Energy.

[9] Su C., Dalgren J., Palm B. (2021). High-resolution mapping of the clean heat sources for district heating in Stockholm City. Energy Convers. Manag..

[10] (2020). Taking the Next Steps: Stockholm, the Circular City, DatacenterDynamics. https://www.datacenterdynamics.com/en/analysis/taking-next-steps-stockholm-circular-city/.

[11] Cho J., Park B., Jeong Y. (2019). Thermal Performance Evaluation of a Data Center Cooling System under Fault Conditions. Energies.

[12] Marcel A., Cristian P., Eugen P., Claudia P., Cioara T., Anghel I., Ioan S. Thermal aware workload consolidation in cloud data centers. Proceedings of the 2016 IEEE 12th International Conference on Intelligent Computer Communication and Processing (ICCP).

[13] Cho J., Woo J., Park B., Lim T. (2020). A Comparative CFD Study of Two Air Distribution Systems with Hot Aisle Containment in High-Density Data Centers. Energies.

[14] Koronen C., Åhman M., Nilsson L.J. (2020). Data centres in future European energy systems—Energy efficiency, integration and policy. Energy Effic..

[15] Silva-Llanca L., del Valle M., Ortega A., Díaz A.J. (2019). Cooling Effectiveness of a Data Center Room under Overhead Airflow via Entropy Generation Assessment in Transient Scenarios. Entropy.

[16] Antal M., Pop C., Cioara T., Anghel I., Salomie I., Pop F. (2020). A system of systems approach for data centers optimization and integration into smart energy grids. Future Gener. Comput. Syst..

[17] Oltmanns J., Sauerwein D., Dammel F., Stephan P., Kuhn C. (2020). Potential for waste heat utilization of hot-water-cooled data centers: A case study. Energy Sci. Eng..

[18] Swinhoe D. (2021). Switch Datacenters to Heat Homes and Offices Using Residual Server Heat, Data Center Dynamics. https://www.datacenterdynamics.com/en/news/switch-datacenters-heat-homes-and-offices-using-residual-server-heat/.

[19] Wheatcroft E., Wynn H., Lygnerud K., Bonvicini G., Leonte D. (2020). The Role of Low Temperature Waste Heat Recovery in Achieving 2050 Goals: A Policy Positioning Paper. Energies.

[20] Jirinec J., Rot D. The Control System for Heating of Small Buildings with Heat Recovery unit and Heat Pump. Proceedings of the 2020 21st International Scientific Conference on Electric Power Engineering (EPE).

[21] Härtel P., Ghosh D. (2020). Modelling heat pump systems in low-carbon energy systems with significant cross-sectoral integration. IEEE Trans. Power Syst..

[22] Wang J., Zhong H., Tan C.W., Chen X., Rajagopal R., Xia Q., Kang C. (2018). Economic Benefits of Integrating Solar-Powered Heat Pumps into a CHP System. IEEE Trans. Sustain. Energy.

[23] Garofalo E., Bevione M., Cecchini L., Mattiussi F., Chiolerio A. (2020). Waste Heat to Power: Technologies, Current Applications, and Future Potential. Energy Technol..

[24] Ebrahimi K., Jones G.F., Fleischer A.S. (2015). Thermo-economic analysis of steady state waste heat recovery in data centers using absorption refrigeration. Appl. Energy.

[25] Antal M., Cioara T., Anghel I., Pop C., Salomie I., Bertoncini M., Arnone D. DC Thermal Energy Flexibility Model for Waste Heat Reuse in Nearby Neighborhoods. Proceedings of the Eighth International Conference on Future Energy Systems (ACM e-Energy ’17).

[26] Dayarathna M., Wen Y., Fan R. (2016). Data Center Energy Consumption Modeling: A Survey. IEEE Commun. Surv. Tutor..

[27] Matko V., Brezovec B., Milanovič M. (2019). Intelligent Monitoring of Data Center Physical Infrastructure. Appl. Sci..

[28] Rastegarpour S., Caseri L., Ferrarini L., Gehrke O. Experimental Validation of the Control-Oriented Model of Heat Pumps for MPC Applications. Proceedings of the 2019 IEEE 15th International Conference on Automation Science and Engineering (CASE).

[29] Grishina A., Chinnici M., Kor A.-L., Rondeau E., Georges J.-P. (2020). A Machine Learning Solution for Data Center Thermal Characteristics Analysis. Energies.

[30] Durand-Estebe B., le Bot C., Mancos J.N., Arquis E. (2013). Data center optimization using PID regulation in CFD simulations. Energy Build..

[31] Kheirabadi A.C., Groulx D. (2016). Cooling of server electronics: A design review of existing technology. Appl. Therm. Eng..

[32] Li Y., Wang X., Luo P., Pan Q. (2019). Thermal-Aware Hybrid Workload Management in a Green Datacenter towards Renewable Energy Utilization. Energies.

[33] Daniels S.J., Rahat A.A.M., Everson R.M., Tabor G.R., Fieldsend J.E., Auger A., Fonseca C., Lourenço N., Machado P., Paquete L., Whitley D. (2018). A Suite of Computationally Expensive Shape Optimisation Problems Using Computational Fluid Dynamics. Parallel Problem Solving from Nature, Proceedings of the PPSN 2018: Parallel Problem Solving from Nature–PPSN XV, Coimbra, Portugal, 8–12 September 2018.

[34] Lye K.O., Mishra S., Ray D. (2020). Deep learning observables in computational fluid dynamics. J. Comput. Phys..

[35] Jonas M., Gilbert R.R., Ferguson J., Varsamopoulos G., Gupta S. A transient model for data center thermal prediction. Proceedings of the 2012 International Green Computing Conference, IGCC 2012.

[36] Berezovskaya Y., Yang C., Mousavi A., Vyatkin V., Minde T.B. (2020). Modular Model of a Data Centre as a Tool for Improving Its Energy Efficiency. IEEE Access.

[37] Wang F., Huang Y., Prasetyo B. (2019). Energy-Efficient Improvement Approaches through Numerical Simulation and Field Measurement for a Data Center. Energies.

[38] Sánchez C., Bloch L., Holweger J., Ballif C., Wyrsch N. (2019). Optimised Heat Pump Management for Increasing Photovoltaic Penetration into the Electricity Grid. Energies.

[39] Li L., Liang C., Liu J., Nath S., Terzis A., Faloutsos C. ThermoCast: A cyber-physical forecasting model for data centers. Proceedings of the ACM SIGKDD International Conference on Knowledge Discovery and Data Mining.

[40] Kumar V.A. (2013). Real Time Temperature Prediction in a Data Center Environment Using an Adaptive Algorithm. Master’s Thesis.

[41] Sharma M., Garg R. (2020). An artificial neural network based approach for energy efficient task scheduling in cloud data centers. Sustain. Comput. Inform. Syst..

[42] Sasakura K., Aoki T., Komatsu M., Watanabe T. (2020). Rack Temperature Prediction Model Using Machine Learning after Stopping Computer Room Air Conditioner in Server Room. Energies.

[43] Ilager S., Ramamohanarao K., Buyya R. (2021). Thermal Prediction for Efficient Energy Management of Clouds Using Machine Learning. IEEE Trans. Parallel Distrib. Syst..

[44] Zapater M., Risco-Martín J.L., Arroba P., Ayala J., Moya J., Hermida R. (2016). Runtime Data Center Temperature Prediction using Grammatical Evolution Techniques. Appl. Soft Comput..

[45] Brenner P., Go D.B., Buccellato A.P.C. Data Center Heat Recovery Models and Validation: Insights from Environmentally Opportunistic Computing. Proceedings of the ASHRAE Winter Conference Technical Program.

[46] Wang S.J., Jin C.Z., Liu G., Tan V., Han X. (2006). An extension of earley’s algorithm for extended grammars. Computational Methods.

[47] Akbari A., Khonsari A., Ghoreyshi S.M. (2020). Thermal-Aware Virtual Machine Allocation for Heterogeneous Cloud Data Centers. Energies.

[48] Chaudhry M.T., Ling T.C., Manzoor A., Hussain S.A., Kim J. (2015). Thermal-Aware Scheduling in Green Data Centers. ACM Comput. Surv..

[49] Cioara T., Antal M., Antal C.D., Anghel I., Bertoncini M., Arnone D., Lazzaro M., Mammina M., Velivassaki T.-H., Voulkidis A. (2020). Data Centers Optimized Integration with Multi-Energy Grids: Test Cases and Results in Operational Environment. Sustainability.

[50] Yao J., Guan H., Luo J., Rao L., Liu X. (2015). Adaptive Power Management through Thermal Aware Workload Balancing in Internet Data Centers. IEEE Trans. Parallel Distrib. Syst..

[51] Moore J., Chase J., Ranganathan P., Sharma R. Making scheduling “cool”: Temperature-aware workload placement in data centers. Proceedings of the Annual Conference on USENIX Annual Technical Conference (ATEC ’05).

[52] Singh S., Chana I. (2016). A Survey on Resource Scheduling in Cloud Computing: Issues and Challenges. J. Grid Comput..

[53] Yang J., Xiao W., Jiang C., Hossain M.S., Muhammad G., Amin S.U. (2019). AI-Powered Green Cloud and Data Center. IEEE Access.

[54] Wang L., von Laszewski G., Huang F., Dayal J., Frulani T., Fox G. (2011). Task scheduling with ANN-based temperature prediction in a data center: A simulation-based study. Eng. Comput..

[55] Nejad S.M.M., Badawy G., Down D.G. (2021). Holistic thermal-aware workload management and infrastructure control for heterogeneous data centers using machine learning. Future Gener. Comput. Syst..

[56] Van Damme T., de Persis C., Tesi P. (2017). Optimized Thermal-Aware Job Scheduling and Control of Data Centers. IFAC-Pap..

[57] Ni J., Jin B., Zhang B., Wang X. (2017). Simulation of Thermal Distribution and Airflow for Efficient Energy Consumption in a Small Data Centers. Sustainability.

[58] Sun H., Stolf P., Pierson J. (2017). Spatio-temporal thermal-aware scheduling for homogeneous high-performance computing datacenters. Future Gener. Comput. Syst..

[59] Kumar M., Sharma S.C. (2017). Dynamic load balancing algorithm for balancing the workload among virtual machine in cloud computing. Procedia Comput. Sci..

[60] Zhou J., Yan J., Chen J., Wei T. (2016). Peak Temperature Minimization via Task Allocation and Splitting for Heterogeneous MPSoC Real-Time Systems. J. Signal Process. Syst..

[61] Anghel I., Cioara T., Salomie I., Copil G., Moldovan D., Pop C. Dynamic frequency scaling algorithms for improving the CPU’s energy efficiency. Proceedings of the 2011 IEEE 7th International Conference on Intelligent Computer Communication and Processing.

[62] Liu H., Liu B., Yang L.T., Lin M., Deng Y., Bilal K., Khan S.U. (2018). Thermal-Aware and DVFS-Enabled Big Data Task Scheduling for Data Centers. IEEE Trans. Big Data.

[63] Cioara T., Salomie I., Anghel I., Chira I., Cocian A., Henis E., Kat R., Maximilien E.M., Rossi G., Yuan S.T., Ludwig H., Fantinato M. (2011). A Dynamic Power Management Controller for Optimizing Servers’ Energy Consumption in Service Centers. Service-Oriented Computing, Proceedings of the International Conference on Service-Oriented Computing ICSOC 2010, Stockholm, Sweden, 23–27 November 2010.

[64] Li X., Xie N., Tian X. (2017). Dynamic Voltage-Frequency and Workload Joint Scaling Power Management for Energy Harvesting Multi-Core WSN Node SoC. Sensors.

[65] Fang Q., Wang J., Zhu H., Gong Q. (2014). Using Model Predictive Control in Data Centers for Dynamic Server Provisioning. IFAC Proc. Vol..

[66] Wang J., Shen T., Zhao J., Ma S., Rao W., Zhang Y. Data-driven thermal efficiency modeling and optimization for reheating furnace based on statistics analysis. Proceedings of the 2015 34th Chinese Control Conference (CCC).

[67] Liu J., Goraczko M., James S., Belady C., Lu J., Whitehouse K. The Data Furnace: Heating Up with Cloud Computing. Proceedings of the 3rd USENIX Workshop on Hot Topics in Cloud Computing.

[68] Tang Q., Mukherjee T., Gupta S.K.S., Cayton P. Sensor-based Fast Thermal Evaluation Model for Energy Efficient High-Performance Datacenters. Proceedings of the Fourth International Conference on Intelligent Sensing and Information Processing.

[69] Zhang S., Xia Y. (2018). Solving nonlinear optimization problems of real functions in complex variables by complex-valued iterative methods. IEEE Trans. Cybern..

[70] Belotti P., Kirches C., Leyffer S., Linderoth J., Luedtke J., Mahajan A. (2013). Mixed-integer nonlinear optimization. Acta Numer..

[71] Ruder S. (2016). An overview of gradient descent optimization algorithms. arXiv.

[72] Laabadi S., Naimi M., el Amri H., Boujemâa A. (2018). The 0/1 Multidimensional Knapsack Problem and Its Variants: A Survey of Practical Models and Heuristic Approaches. Am. J. Oper. Res..

[73] Pardey Z., Demetriou D., Erden H., Vangilder J., Khalifa H., Schmidt R. (2015). Proposal for standard compact server model for transient data center simulations. ASHRAE Trans..

[74] Qarnot Datacenter. https://qarnot.com/.

[75] SciPY Optimizer. https://docs.scipy.org/doc/scipy/reference/optimize.html.

[76] SciKit Learn. https://scikit-learn.org/stable/.

[77] Keras. https://keras.io/.

[78] Tensorflow. https://www.tensorflow.org/.

[79] XGBoost. https://xgboost.readthedocs.io/en/latest/.

[80] Heating Blog. https://www.electricpoint.com/heating/electric-heating/how-to-calculate-kw-required-to-heat-a-room.

[81] Intel® Core™ i7-6950X Study. https://www.anandtech.com/show/10337/the-intel-broadwell-e-review-core-i7-6950x-6900k-6850k-and-6800k-tested-up-to-10-cores/10.

